# Army Drift

**Published:** 1898-11

**Authors:** 


### ARMY DRIFT.

Every veterinarian in the Northwest should urge strongly the needs of the profession in our army, and a word to the Secretary of War direct, or through his Congressman, will not be amiss.

One of the experts sent to Tampa to investigate the existence of glanders hailed from New York State, and his chief occupation during the past two or three years was the selling of horse- and cattle-food, and doing the rural districts peddling soaking boots, which he hung as decorations to the bows of his carriage as he made his pilgrimages among the farmers.

Surely after so many years' service, and now an added experience and trials of a semi-tropical climate, should entitle several of our army veterinarians to retirement and a pension. Alas! because they are not commissioned, there is no such provision for them, save by virtue of legislation.

Huntsville, Ala., is now the centre of proposed movements of a number of our army colleagues who are daily expecting orders to embark for Cuba.

Shoulder-straps were in marked evidence at Tampa, and their wearers, clothed with arbitary power, saw no danger in horses condemned for glanders drinking from the same troughs as those marked as free from the disease. Do you wonder at educated and trained men resigning?

Unsound horses, unadapted for the service, were purchased by hundreds by commissioned officers wholly ignorant of the question of the actual needs of those in the service.

There was no place for any one in the Army Veterinary Department but subservient tools to those who were profiting by the decorations that indicated power.

Distemper and glanders have claimed many victims among the horses and mules, and with an ignoring of every salutary sanitary law, competent veterinarians driven out of the service because their lack of authority making it impossible to avoid these results.

"Brothers—Phys. kinly remember me not to Disturbe my medicines"—a leading appointee's notice posted at his medicine supply closet while temporarily absent.

Regimental quartermasters would not accept the responsibility of taking veterinary supplies into the field. A report reached us that there was but one veterinarian at Chickamauga or Tampa with anything approaching a surgical equipment. He became a borrowing centre for such trifles as needles and thread for the care of wounds and injuries. Will not the commission extend its inquiries anent the suffering of the quadruped division of our army service?

The humane societies of our land should join in one solemn, serious protest against the criminal neglect of those animals so essential to the rapid mobilization of our army and so potential in our victories at San Juan and Santiago. They deserve more merciful consideration.

Think of hundreds of valuable animals being sacrificed unto death by the external application of red tape.

Think of a so-called expert veterinarian arriving at Tampa for the purpose of saving some of the recklessly squandered money of our people, announcing his arrival and official mission as that of determining whether the Government's horses were affected with "bacillius malleus" or "tuberculosis."

Nearly all veterinary appointments at Southern posts of the regular army were in direct violation of the spirit of the law governing such selections, and in the face of the letter of the law, that all army veterinarians should be graduates of legally organized and recognized veterinary colleges. A number of those appointed possessed no degree whatever.

One of the most important appointments at the most critical juncture at Tampa was an obscure non-graduate with absolutely no practical knowledge of the work he was to do. Surely the salary-grabbers knew a good thing when they saw it, and politicians mended weak points in their political fences by securing these plums for their division heelers and shouters.

If the army veterinarians at our Southern points had only the rank of second lieutenant they would not have been overridden by the cavalry and quartermaster officers, and our people saddled with an enormous waste of money, unnecessary suffering of hundreds of animals, and death from neglect.

Veterinarians are willing to sustain every responsibility placed upon them as a profession when such a system of examination is inaugurated as that under the Civil Service Commission for the Department of Agriculture, but not when there is a complete disregard of every test as to fitness and partisan appointments are made by spoilsmen.

One branch of the cavalry located at Tampa received two hundred and fifty remounts; over one-half of these were sick with strangles, influenza, glanders. Tied on the same lines, watered from the same troughs—could there be aught else but serious results? Even the corrals, such as they were, providing some safeguards, were ignored by officers, in orders given for animals.

A failure upon the part of officers in power at Tampa and Jacksonville to accept competent veterinary advice is largely the cause of the widespread existence of glanders at those posts.

Come got 3 mules out of my pen

Dr. ———

Wagonmaster for —— Infantry.

A sample of the intelligence of one of the veterinary appointees in Florida in reporting to the Quartermaster-General's office.

Over eight thousand horses and mules at the Tampa corral for over a month without competent veterinary attention! The responsibility for this blunder, almost criminal in character, should be investigated and severe punishment meted out to those responsible. The presence of glanders is an unnecessary danger to our livestock interests as well as to every army officer and soldier brought in contact with this dangerous pest of the equine species.

Political influence and pressure of those who seek preferment on other lines than merit, at a critical hour has pervaded the veterinary service sought for at our Southern army posts, and ignorance and hesitation when and how to act are now adding to the terrible losses among the horses and mules. How long is this condition to continue?

A profession that has demonstrated its worth and efficiency through a proper recognition in the Department of Agriculture is demoralized and palsied in our army department for want of a similar recognition with power to act. Criminal blundering seems to have been the order of the hour. We would like to hear from the Quartermaster-General's Department on this score. Let the Investigating Committee add this to their field of research as to who have so fearfully blundered.

An ignorant pretender among veterinarians was sent to Tampa to avoid being ordered to Cuba, and, as if to add to his utter helplessness, he was placed there without medicines, instruments, or assistants. His duty finally resolved itself into that of discriminating between the dead and living, and seeing that the former were dragged out.

Almost every appointment of veterinarians to our recent Southern army posts by the Quartermaster-General's office was through political influence and a part and parcel of the "infamous spoils system" that has degraded every branch of the public service where it prevails or has prevailed.

Tenure of office has no security under appointments gained by "political pull," as was learned by one of the veterinarians recently relieved from duty at Tampa and who claimed to have as his backer no less a personage than Mark Hanna.

Speed the outgoing, welcome the incoming, was the order of the day at Tampa about November 1st. The cry for retrenchment of expenses, by virtue of which several veterinarians were relieved from duty, seems also to have marked the time for the arrival of several new appointees whose backers with political influence had finally landed their constituents in public office.

